# Ophthalmology as Related to General Medicine

**Published:** 1878-08

**Authors:** E. L. Holmes


### OPHTHALMOLOGY AS RELATED TO GENERAL MEDICINE.

By E. L. Holmes, M. D.

*(Read at the last meeting of the Illinois State Medical Society.)*

The best text books on ophthalmology, especially in German, English and French, as compared with those on other branches of medicine and surgery, possess a very high degree of merit.

The relative difficulties which confront the student in the study of these different works, may, I think, be stated somewhat as follows :

A fair knowledge of ophthalmic and of general medical science may be acquired by any one of moderate capacity, who commences study with an elementary knowledge of Latin and Greek and of physics.

Unfortunately, too many medical students not only possess meager knowledge of these branches but, to use a common expression, "do not know certainly what little they do know."

Commencing on the low plane of mental discipline, which I have just mentioned, the diligent student may, nevertheless, pursue his studies with no special difficulty in ophthalmology, till he enters upon the study of the ophthalmoscope and of refraction and accommodation. Here the difficulties for nearly all medical students are very great.

It may be said in truth, I think, that good general surgeons are liable to fail in ophthalmic surgery not so much from want of special delicacy in the use of their fingers, as from want of practice with fine instruments and the lack of judgment founded on frequent review of fundamental principles.

Good physicians also for a similar reason often fail to diagnosticate and to treat properly common diseases of the eye.

I may, without danger of making odious comparisons between the general practitioner and the oculist, call attention to several points, wherein the former is liable to injure patients by not comprehending a few principles *well.*

Consider for a moment the subject of glaucoma. While many physicians still utterly fail to recognize glaucoma, many more than formerly observe cases of the disease at eye clinics and thus learn to comprehend this condition of the eye when they meet it. They are aware that the eye is glaucomatous, when suddenly or slowly there appears an abnormal hardness of the globe, with an enlarged pupil, pain and persistent colored rings around an artificial light at night. They are aware also that an operation, usually iridectomy is most generally advised.

Still, intelligent physicians often advise patients suffering from the terrible pains of acute glaucoma, to undergo a course of internal treatment before seeking relief by an operation.

Every physician should be able to detect an increase of hardness of the globe and should urge, without delay, the appropriate operation when it is a marked symptom.

Iritis is a disease, which every physician should recognize in its early stages. Yet we find the *application* of knowledge often at fault.

Typical cases are easily diagnosticated. There is frequently a failure to make a positive and early diagnosis by neglecting to demonstrate the irregularity and immobility of the pupil after the instillation of a drop or two of solution of atropine upon the conjunctiva. Many eyes are partially or some totally lost in consequence of this omission.

The chapter on sympathetic ophthalmitis is of so very great practical importance to the general practitioner that it should never be neglected by him. We should constantly impress upon his mind the few great pripciples which govern the action of the enlightened surgeon in the management of certain injuries of the globe. These principles in general are simple and readily understood.

The difficulty lies in the fact that practitioners are too often in blank ignorance of these principles. They may have listened to a statement of the facts, presented in a lecture or two, but ever after fail to study and review the subject. The consequence is hundreds of patients with an injury of one eye become totally blind from sympathetic inflammation of the other eye, when perfect vision of the uninjured eye might have been saved.

I have frequently alluded to this subject before this Association. Its importance, and the fact that it has in recent times called forth numerous and earnest papers from many of the most celebrated writers in ophthalmic science in every portion of the world, is sufficient apology for again briefly occupying the time of the society.

Another subject of very great importance has occupied the attention of the most celebrated ophthalmologist, for years. The general results of these observations, as expressed in simple language, should be deeply impressed on the mind of the general practitioner. I refer to ophthalmic hygiene. The subject in its relation to the adaptation of glasses is very difficult for most students and practitioners to master. It requires long study and ample experience to overcome these difficulties. And yet physicians, educated in a few fundamental principles alone, are capable of doing very great good in their fields of labor.

It should be known that, while patients with uncomplicated myopia and presbyopia *may* select their own spectacles without danger, in very many instances they may do great harm. As a rule, it is safe to state that whenever ordinary work with the eyes, assisted or not by spectacles, causes discomfort, great care and skill is often requisite in selecting the proper lenses.

No physician should be ignorant of the fact that a high degree of myopia, especially if progressive, may endanger vision by careless usage.

Certain facts regarding myopia should in no wise be confined to a specialty.

A physician to whom is entrusted the care of a family's health, should always be able and ready to give good advice regarding the necessity of having the family sewing and reading room well lighted by day, and in the evening well supplied with artificial light. Shaded rooms and those lighted with dull lamps or duller candles are no places for those using their eyes on close work. Children should be taught to avoid studying too far from the source of light in any room. Both excessively strong lights and cross lights should be avoided, as also too fine or indistinct print. The habit of bending the face too much forward while reading should never be encouraged.

Physicians in every town and village should interest themselves in the structure and lighting of school houses, in the form of desks, and should advise teachers to have all possible regard for the welfare of the children under their care, in reference to the use and abuse of their eyes.

Every physician should possess the skill to use the ophthalmoscope intelligibly in the examination of the anterior structures of the eyes.

The condition of the pupillary edge of the iris, of the lens and the anterior portion of the vitreous, can be quite readily determined by a little practice with the instrument.

In this way the physician can settle authoritatively many questions regarding the conditions of an eye which he could not otherwise comprehend.

On the other hand, an intelligent examination of the choroid, retina and optic nerve, and of refraction by means of the ophthalmoscope, requires such long and patient training that very few physicians in general practice can ever hope to overcome the difficulties.

Fortunately the cases are comparatively few in which the practitioner can gain much assistance from the ophthalmoscope regarding a patient's general health. In the vast majority of cases the instrument reveals simply the condition of the fundus of the globe and nothing definite beyond it.

It is true that not unfrequently, after an examination of the eye, the examiner almost intuitively enquires whether the patient is suffering from Bright's disease, diabetes, syphilis, tumor in the brain, or whether he is under the chronic influence of alcohol or tobacco. In nearly all these cases, however, the careful physician is able to discover the presence of these diseases before an ophthalmoscopic examination is made. A great mass of supposed facts have been accumulated by distinguished neurologists, which are believed by some to prove that the condition of the vascular supply of the brain may be determined by ophthalmoscopic examinations of the retina and optic nerve disc. But these observations have not yet, I think, been satisfactorily analyzed to enable us to form an accurate diagnosis of the cerebral tissues by such examinations.

I think there can be no dissent from the proposition, that the study of ophthalmology as a specialty should be commenced only after the study of medicine has been pursued at least three years.

And yet every physician upon whom those afflicted with diseases of the eye are absolutely dependent for aid, should study and review year after year some of the fundamental principles of ophthalmology.

A few facts regarding some of the most important diseases should be stated as apothegms, and for ever fixed in the memory.

All this touches very closely upon the whole question of medical education. If a student has faithfully studied and learned the few principles alone in ophthalmology which I have enumerated, he will have required more time than students usually can devote to this branch. If he has pursued each of the other branches of medicine in the same careful, but may be superficial way, trusting to the future for the perfection of his education, he has a task which every medical student should perform—and yet which the average medical student in America cannot perform in the three years of his course.

It is much better that studies should be systematized, than that they should be pursued without system. And yet with the very best of systematic arrangement, very few students can prepare themselves for the proper practice of their profession in three years.

I believe public opinion will compel the leading medical schools of this country to elevate the standard necessary for graduation. I am thoroughly convinced, however, that no real advance will be made before the schools insist upon *four full* years of study, each year being divided into two regular terms of at least four and a half months each.

An equivalent to this change would be, perhaps, the requirement that no student should be admitted to a medical college as a matriculate, who had not at least received as complete an education as can be obtained in a good English high-school, and has not studied quite thoroughly chemistry and the use of the microscope, with microscopic anatomy.

I believe the profession, public opinion and the medical colleges themselves will gradually effect a change for the better in the results of medical teaching. I believe, however, that custom, the manner in which American laws are enacted and enforced, the habit in this country of endeavoring to do things rapidly, if not thoroughly, the plan on which our medical colleges are founded and supported as compared with European medical colleges or even with our own literary institutions, will render any absolute radical reform exceedingly slow.

